# Dilead(II) chromium(III) hepta­fluoride

**DOI:** 10.1107/S1600536810011608

**Published:** 2010-04-10

**Authors:** Armel Le Bail

**Affiliations:** a Université du Maine, Laboratoire des Oxydes et Fluorures, CNRS UMR 6010, Avenue O. Messiaen,72085 Le Mans, France

## Abstract

Single crystals of the title compound, Pb_2_CrF_7_, were obtained by solid-state reaction. The monoclinic structure is isotypic with Pb_2_RhF_7_ and is built up of CrF_6_^3−^ octa­hedra isolated from each other, inserted in a fluorite-related matrix of PbF_6_ distorted octa­hedra, and PbF_8_ square anti­prisms sharing edges and corners. The seventh F atom is ‘independent’, connected only to three Pb atoms within FPb_3_ triangles, sharing an edge and building an almost planar Pb_4_F_2_ unit, so that the formula can alternatively be written as Pb_2_F(CrF_6_).

## Related literature

For the Pb_2_RhF_7_ structure-type, see: Domesle & Hoppe (1983 ▶), and for isostructural Sr_2_RhF_7_, see: Grosse & Hoppe (1987 ▶). For the indexed powder pattern of Pb_2_CrF_7_, see: de Kozak *et al.* (1999 ▶). For other compounds containing ‘independent’ fluorine atoms coordinated to three cations (Ca or Sr) in a plane, see: Ca_2_AlF_7_ (Domesle & Hoppe, 1980 ▶); Sr_5_Zr_3_F_22_ (Le Bail, 1996 ▶); Sr_5_(VOF_5_)_3_F(H_2_O)_3_ (Le Bail *et al.*, 2009 ▶). For fluorite-related lead-based compounds, see: Pb_8_MnFe_2_F_24_ (Le Bail & Mercier, 1992 ▶); Pb_2_ZrF_8_ (Le Bail & Laval, 1998 ▶). For the structure simulation of fluoride glasses containing lead by using crystalline models, see: Le Bail (1989 ▶, 2000 ▶). For details and parameters of the bond-valence model, see: Brown & Altermatt (1985 ▶); Brese & O’Keeffe (1991 ▶).

## Experimental

### 

#### Crystal data

Pb_2_CrF_7_
*M* *_r_* = 599.40
Monoclinic, 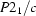

*a* = 5.4626 (7) Å
*b* = 11.2085 (15) Å
*c* = 9.5738 (11) Å
β = 91.197 (10)°
*V* = 586.05 (13) Å^3^
*Z* = 4
Mo *K*α radiation
μ = 59.61 mm^−1^
*T* = 293 K
0.12 × 0.07 × 0.01 mm

#### Data collection

Siemens AED2 diffractometer
Absorption correction: Gaussian (*SHELX76*; Sheldrick, 2008 ▶) *T*_min_ = 0.011, *T*_max_ = 0.321
6770 measured reflections
2506 independent reflections
2082 reflections with *I* > 2σ(*I*)
*R*_int_ = 0.041
3 standard reflections every 120 min  intensity decay: 15%

#### Refinement

*R*[*F* ^2^ > 2σ(*F* ^2^)] = 0.036
*wR*(*F* ^2^) = 0.084
*S* = 1.64
2506 reflections
93 parameters
Δρ_max_ = 3.17 e Å^−3^
Δρ_min_ = −2.20 e Å^−3^

### 

Data collection: *STADI4* (Stoe & Cie, 1998 ▶); cell refinement: *STADI4*; data reduction: *X-RED* (Stoe & Cie, 1998 ▶); program(s) used to solve structure: *SHELXS97* (Sheldrick, 2008 ▶); program(s) used to refine structure: *SHELXL97* (Sheldrick, 2008 ▶); molecular graphics: *DIAMOND* (Brandenburg, 2005 ▶) and *ORTEP-3* (Farrugia, 1997 ▶); software used to prepare material for publication: *publCIF* (Westrip, 2010 ▶).

## Supplementary Material

Crystal structure: contains datablocks I, global. DOI: 10.1107/S1600536810011608/wm2317sup1.cif

Structure factors: contains datablocks I. DOI: 10.1107/S1600536810011608/wm2317Isup2.hkl

Additional supplementary materials:  crystallographic information; 3D view; checkCIF report

## Figures and Tables

**Table 1:** Selected bond lengths (Å)

Pb1—F1	2.324 (5)
Pb1—F7^i^	2.437 (5)
Pb1—F4^ii^	2.439 (5)
Pb1—F5^iii^	2.620 (6)
Pb1—F6^iv^	2.640 (7)
Pb1—F2^v^	2.899 (6)
Pb1—F6^v^	3.067 (7)
Pb2—F1	2.412 (5)
Pb2—F1^ii^	2.441 (4)
Pb2—F5^i^	2.497 (6)
Pb2—F3^vi^	2.512 (6)
Pb2—F2^iv^	2.586 (5)
Pb2—F6	2.632 (6)
Pb2—F3^iv^	2.653 (5)
Pb2—F7	2.743 (6)
Cr—F2	1.883 (5)
Cr—F3	1.900 (5)
Cr—F4	1.907 (5)
Cr—F5	1.909 (5)
Cr—F6	1.912 (6)
Cr—F7	1.931 (4)

Symmetry codes: (i) 
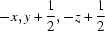
; (ii) 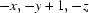; (iii) 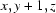; (iv) 
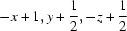
; (v) 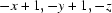; (vi) 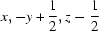.

**Table 2:** Valence-bond analysis according to the empirical expression from Brown & Altermatt (1985 ▶), using parameters for solids from Brese & O’Keeffe (1991 ▶), with two results for Pb1 and Pb2 according to their coordinations (respectively VI or XI, and VIII or IX)

	Cr	Pb1	Pb2	Σ	Σexpected
F1		0.45	0.36 + 0.33	1.14	1
F2	0.52	0.10 + 0.05	0.22	0.89	1
F3	0.50	0.04	0.27 + 0.19	1.00	1
F4	0.49	0.33 + 0.04	0.05	0.91	1
F5	0.48	0.20	0.28	0.96	1
F6	0.48	0.19 + 0.06	0.20	0.93	1
F7	0.46	0.33 + 0.03	0.15	0.97	1
Σ	2.93	1.60 (VI)	2.00 (VIII)		
or		1.82 (XI)	2.05 (IX)		
Σexpected	3	2	2		

**Table 3:** Comparison of the cell parameters of the three isostructural hepta­fluorides showing a noticeable anomaly on the *c* parameter of the chromium compound

Formula	*a*	*b*	*c*	β	*V*
Pb_2_RhF_7_*^*a*^*	5.569	11.854	8.832	91.00	582.96
Sr_2_RhF_7_*^*b*^*	5.510	11.628	8.640	90.98	553.49
Pb_2_CrF_7_*^*c*^*	5.463	11.208	9.574	91.20	586.05

Notes: (*a*) Domesle & Hoppe (1983 ▶); (*b*) Grosse & Hoppe (1987 ▶); (*c*) this work.

**Fig. 1.**
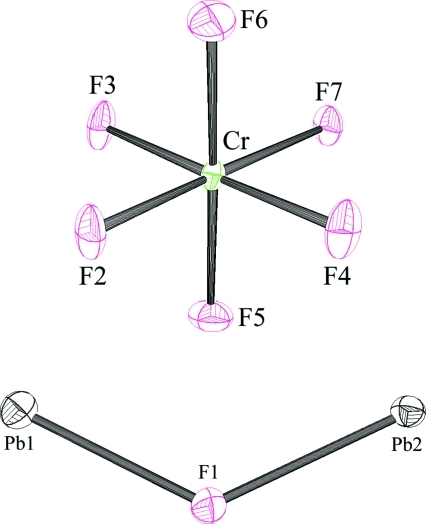
ORTEP-3 view (Farrugia, 1997) of the regular [CrIIIF6]3- octahedron and of the "independent" fluoride ion F1 connected to Pb1 and Pb2 completing the Pb2F(CrF6) formula (ellipsoids at the 50% probability level).

**Fig. 2.**
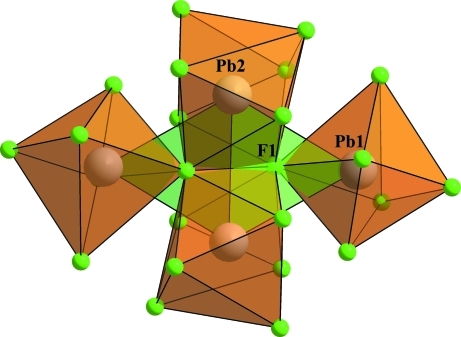
Diamond (Brandenburg, 2005) view of the two F1Pb3 triangles sharing an edge in order to form the planar F2Pb4 unit with Pb1F6 distorted octahedra and Pb2F8 square antiprisms.

**Fig. 3.**
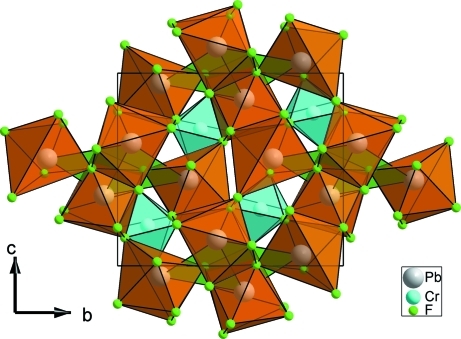
Diamond (Brandenburg, 2005) view of the crystal packing along [100] showing the isolated [CrF6] octahedra,

**Fig. 4.**
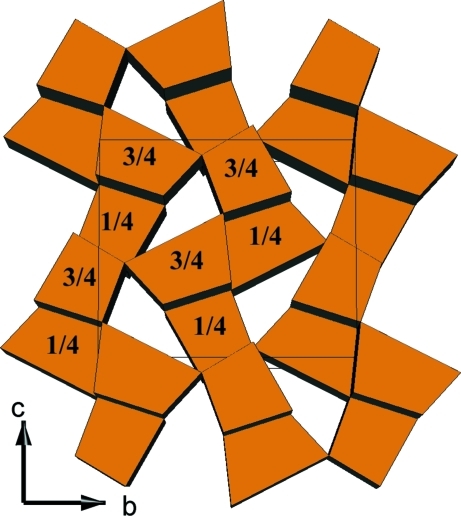
Idealized (with small displacement of some F atoms) view of the Pb atoms alternating positions close to x = 1/4 and 3/4, building corrugated strips with the PbF2 fluorite structure. Similar kinked blocks were observed for Pb2ZrF8 (Le Bail & Laval, 1998).

## supplementary crystallographic information

### Comment

Continuing investigations of crystalline complex compounds of lead and 3*d* metal cation fluorides having chemical formulations similar to some fluoride glasses for structure modelling purposes (Le Bail, 2000), such as Pb_8_MnFe_2_F_24_ (Le Bail & Mercier, 1992) or NaPbFe_2_F_9_ (Le Bail, 1989), the title compound, Pb_2_CrF_7_, was synthesized and characterized from single crystal X-ray data. The results confirm that it is isostructural with Pb_2_RhF_7_ (Domesle & Hoppe, 1983), as mentioned previously by de Kozak *et al.* (1999). The monoclinic structure is built up of CrF_6_^3-^ octahedra (Fig. 1) isolated from each other, inserted in a fluorite-related matrix of PbF_6_ distorted octahedra and PbF_8_ square antiprisms sharing edges and corners. The seventh fluorine atom is "independent", connected only to 3 Pb atoms in FPb_3_ triangles, sharing an edge and building an almost planar Pb_4_F_2_ unit (Fig. 2), so that the formula can be alternatively written as Pb_2_F(CrF_6_). There are some differences observed with the Pb_2_RhF_7_ structure-type. The Pb1 atom appears to be six-coordinated in Pb_2_CrF_7_ with Pb—F distances ranging from 2.324 (5) to 2.899 (6) Å, there are then 5 next F atoms being between 3.067 (7) and 3.364 (6) Å whereas in Pb_2_RhF_7_, Pb1 looks eightfold coordinated (Pb—F distances between 2.375 and 2.744 Å), with two next F atoms at 3.105 and 3.219 Å. The bond valence calculations show that the first 6 F atoms around Pb1 cannot really satisfy the Pb^2+^ charge and that the 5 next F atoms contribute to the overall bond valence (Table 2). The behaviour of the cell parameters is surprising in the series of the three isostructural compounds. If all cell parameters are logically smaller in Sr_2_RhF_7_ (Grosse & Hoppe, 1987) than in Pb_2_RhF_7_, due to the smaller Sr^2+^ size, in Pb_2_CrF_7_ one observes that only *a* and *b* are smaller but that *c* is much larger, in spite of the Cr^3+^ cation being smaller than Rh^3+^ (Table 3). Finally, the cell volume of Pb_2_CrF_7_ is even slightly larger than that of Pb_2_RhF_7_.

Usually, such fluorinated phases with high *A*^II^/*M* ratio (*A*^II^ = Ca, Pb, Sr ; *M* = 3*d* element or In, Nb, Zr, etc), are found to be related to the fluorite structure adopted by PbF_2_ or SrF_2_. The relation is in general easy to establish due to the presence of "independent" F atoms (not bonded to *M*) which are found to form characteristic FPb_4_ tetrahedra. But this is not obvious here (Fig. 3) since no such FPb_4_ tetrahedron is observed. Other compounds containing "independent" fluorine atoms coordinated to three cations (Ca or Sr) in a plane are Ca_2_AlF_7_ (Domesle & Hoppe, 1980), Sr_5_Zr_3_F_22_ (Le Bail, 1996) and Sr_5_(VOF_5_)_3_F(H_2_O)_3_ (Le Bail *et al.*, 2009), the last two being strongly related to the fluorite structure in which the FSr_3_ triangles were found to interconnect the fluorite-related blocks. An examination of the Pb coordinates in Pb_2_CrF_7_ along the *a* axis (which is close to the PbF_2_ fluorite cell parameter) shows that they alternate at values *x*~1/4 and 3/4, forming fluorite-related strips corrugating in the *ac* plane where highly distorted PbF_8_ cubes as expected in the fluorite structure were obtained by small displacements of some of the F atoms as evidenced in Fig. 4 . Similar corrugated strips were observed for the Pb_2_ZrF_8_ structure (Le Bail & Laval, 1998), where the Zr^4+^ cations occupy bicapped trigonal prisms at positions similar to those of Cr^3+^ in the title compound. In Pb_2_ZrF_8_, the Pb^2+^ lone pair position was suggested by comparison of the Pb coordination with that of Ba in the isostructural α-Ba_2_ZrF_8_ structure, observing some strong distorsions, the stereochemichally active lone pair repelling clearly some F atoms. The same exercice is not so obvious here, even by comparing Sr_2_RhF_7_ with Pb_2_RhF_7_. However, there is a specific pattern of differences in the bond lengths (3 adjacent short Pb—F bonds and 3 adjacent long ones for Pb1; 4 short and 4 long for Pb2) which could be attributed to repulsion effects involving a somehow weak stereochemically active lone pair producing the longer Pb—F distances. The Pb1 lone pair is thus probably oriented towards the barycenter of the (F5—F6—F2) face of the Pb1F_6_ octahedron, a similar reasoning applying to the Pb2 lone pair.

### Experimental

Solid state reaction between 2PbF_2_ and CrF_3_ at 773 K for 96 hours in a platinum tube sealed under argon yieded single crystal of the title compound.

### Refinement

The highest residual peak and deepest hole in the final difference map were located respectively 0.67 Å and 0.83 Å from the Pb2 atom.

### Crystal data

**Table:**

Pb_2_CrF_7_	*F*(000) = 1004
*M**_r_* = 599.40	*D*_x_ = 6.793 Mg m^−^^3^
Monoclinic, *P*2_1_/*c*	Mo *K*α radiation, λ = 0.71069 Å
Hall symbol: -P 2ybc	Cell parameters from 40 reflections
*a* = 5.4626 (7) Å	θ = 2.8–35°
*b* = 11.2085 (15) Å	µ = 59.61 mm^−^^1^
*c* = 9.5738 (11) Å	*T* = 293 K
β = 91.197 (10)°	Platelet, green
*V* = 586.05 (13) Å^3^	0.12 × 0.07 × 0.01 mm
*Z* = 4	

### Data collection

**Table:**

Siemens AED2 diffractometer	2082 reflections with *I* > 2σ(*I*)
Radiation source: fine-focus sealed tube	*R*_int_ = 0.041
graphite	θ_max_ = 35.0°, θ_min_ = 2.8°
2θ/ω scans	*h* = −8→8
Absorption correction: gaussian (*SHELX76*; Sheldrick, 2008)	*k* = 0→18
*T*_min_ = 0.011, *T*_max_ = 0.321	*l* = 0→15
6770 measured reflections	3 standard reflections every 120 min
2506 independent reflections	intensity decay: 15%

### Refinement

**Table:**

Refinement on *F*^2^	Primary atom site location: structure-invariant direct methods
Least-squares matrix: full	Secondary atom site location: difference Fourier map
*R*[*F*^2^ > 2σ(*F*^2^)] = 0.036	*w* = [exp(2.00(sinθ/λ)^2^)]/[σ^2^(*F*_o_^2^) + (0.0528*P*)^2^] where *P* = 0.33333*F*_o_^2^ + 0.66667*F*_c_^2^
*wR*(*F*^2^) = 0.084	(Δ/σ)_max_ < 0.001
*S* = 1.64	Δρ_max_ = 3.17 e Å^−^^3^
2506 reflections	Δρ_min_ = −2.20 e Å^−^^3^
93 parameters	Extinction correction: *SHELXL97* (Sheldrick, 2008), Fc^*^=kFc[1+0.001xFc^2^λ^3^/sin(2θ)]^-1/4^
0 restraints	Extinction coefficient: 0.0171 (7)
0 constraints	

### Special details

**Table:**

Geometry. All esds (except the esd in the dihedral angle between two l.s. planes) are estimated using the full covariance matrix. The cell esds are taken into account individually in the estimation of esds in distances, angles and torsion angles; correlations between esds in cell parameters are only used when they are defined by crystal symmetry. An approximate (isotropic) treatment of cell esds is used for estimating esds involving l.s. planes.
Refinement. Refinement of *F*^2^ against ALL reflections. The weighted *R*-factor wR and goodness of fit *S* are based on *F*^2^, conventional *R*-factors *R* are based on *F*, with *F* set to zero for negative *F*^2^. The threshold expression of *F*^2^ > σ(*F*^2^) is used only for calculating *R*-factors(gt) etc. and is not relevant to the choice of reflections for refinement. *R*-factors based on *F*^2^ are statistically about twice as large as those based on *F*, and *R*- factors based on ALL data will be even larger.

### Fractional atomic coordinates and isotropic or equivalent isotropic displacement parameters (Å^2^)

**Table:**

	*x*	*y*	*z*	*U*_iso_*/*U*_eq_	
Pb1	0.21926 (4)	0.81248 (3)	0.05527 (3)	0.01669 (9)	
Pb2	0.23992 (4)	0.43908 (2)	0.13531 (3)	0.01410 (9)	
Cr	0.30002 (17)	0.13284 (9)	0.20773 (11)	0.01105 (17)	
F1	0.1204 (9)	0.6150 (4)	0.0059 (6)	0.0212 (9)	
F2	0.5511 (9)	0.0321 (5)	0.1456 (6)	0.0240 (10)	
F3	0.3978 (9)	0.0941 (5)	0.3938 (6)	0.0233 (10)	
F4	0.2089 (10)	0.1752 (6)	0.0208 (6)	0.0253 (10)	
F5	0.0826 (9)	−0.0003 (5)	0.1978 (7)	0.0251 (11)	
F6	0.5281 (10)	0.2622 (6)	0.2141 (8)	0.0307 (13)	
F7	0.0430 (8)	0.2415 (5)	0.2594 (5)	0.0190 (8)	

### Atomic displacement parameters (Å^2^)

**Table:**

	*U*^11^	*U*^22^	*U*^33^	*U*^12^	*U*^13^	*U*^23^
Pb1	0.01615 (12)	0.01664 (12)	0.01736 (14)	−0.00212 (8)	0.00190 (8)	−0.00028 (8)
Pb2	0.01326 (11)	0.01521 (11)	0.01376 (12)	−0.00018 (7)	−0.00137 (7)	0.00039 (8)
Cr	0.0087 (3)	0.0131 (4)	0.0113 (4)	0.0010 (3)	0.0002 (3)	0.0013 (3)
F1	0.0202 (18)	0.0153 (17)	0.028 (3)	−0.0008 (16)	−0.0086 (17)	0.0004 (17)
F2	0.023 (2)	0.032 (2)	0.018 (2)	0.0174 (19)	0.0007 (17)	−0.0013 (18)
F3	0.023 (2)	0.037 (3)	0.010 (2)	0.0123 (19)	−0.0022 (16)	0.0025 (17)
F4	0.026 (2)	0.039 (3)	0.011 (2)	0.010 (2)	0.0022 (17)	0.0067 (19)
F5	0.023 (2)	0.0195 (19)	0.033 (3)	−0.0058 (18)	0.001 (2)	−0.0061 (19)
F6	0.019 (2)	0.024 (2)	0.049 (4)	−0.0056 (19)	0.000 (2)	0.002 (2)
F7	0.0140 (16)	0.022 (2)	0.021 (2)	0.0084 (16)	0.0022 (15)	−0.0009 (16)

### Geometric parameters (Å, °)

**Table:**

Pb1—F1	2.324 (5)	Cr—F4	1.907 (5)
Pb1—F7^i^	2.437 (5)	Cr—F5	1.909 (5)
Pb1—F4^ii^	2.439 (5)	Cr—F6	1.912 (6)
Pb1—F5^iii^	2.620 (6)	Cr—F7	1.931 (4)
Pb1—F6^iv^	2.640 (7)	F2—F3	2.630 (8)
Pb1—F2^v^	2.899 (6)	F2—F5	2.643 (8)
Pb1—F6^v^	3.067 (7)	F2—F6	2.665 (9)
Pb2—F1	2.412 (5)	F2—F4	2.721 (7)
Pb2—F1^ii^	2.441 (4)	F3—F6	2.659 (9)
Pb2—F5^i^	2.497 (6)	F3—F5	2.734 (8)
Pb2—F3^vi^	2.512 (6)	F3—F7	2.835 (6)
Pb2—F2^iv^	2.586 (5)	F4—F7	2.584 (8)
Pb2—F6	2.632 (6)	F4—F5	2.696 (8)
Pb2—F3^iv^	2.653 (5)	F4—F6	2.698 (9)
Pb2—F7	2.743 (6)	F5—F7	2.784 (8)
Cr—F2	1.883 (5)	F6—F7	2.704 (7)
Cr—F3	1.900 (5)		
			
F2—Cr—F3	88.1 (2)	F4—Cr—F6	89.9 (3)
F2—Cr—F4	91.8 (2)	F5—Cr—F6	177.6 (3)
F3—Cr—F4	178.3 (3)	F2—Cr—F7	176.1 (2)
F2—Cr—F5	88.4 (3)	F3—Cr—F7	95.5 (2)
F3—Cr—F5	91.7 (3)	F4—Cr—F7	84.6 (2)
F4—Cr—F5	89.9 (3)	F5—Cr—F7	92.9 (2)
F2—Cr—F6	89.2 (3)	F6—Cr—F7	89.4 (3)
F3—Cr—F6	88.5 (3)		

Symmetry codes: (i) −*x*, *y*+1/2, −*z*+1/2; (ii) −*x*, −*y*+1, −*z*; (iii) *x*, *y*+1, *z*; (iv) −*x*+1, *y*+1/2, −*z*+1/2; (v) −*x*+1, −*y*+1, −*z*; (vi) *x*, −*y*+1/2, *z*−1/2.

### Table 2 Valence-bond analysis according to the empirical expression from Brown &amp; Altermatt (1985), using parameters for solids from Brese &amp; O'Keeffe (1991), with two results for Pb1 and Pb2 according to their coordinations (respectively VI or XI, and VIII or IX).

**Table:**

	Cr	Pb1	Pb2	Σ	Σexpected
F1		0.45	0.36+0.33	1.14	1
F2	0.52	0.10+0.05	0.22	0.89	1
F3	0.50	0.04	0.27+0.19	1.00	1
F4	0.49	0.33+0.04	0.05	0.91	1
F5	0.48	0.20	0.28	0.96	1
F6	0.48	0.19+0.06	0.20	0.93	1
F7	0.46	0.33+0.03	0.15	0.97	1
Σ	2.93	1.60(VI)	2.00(VIII)		
or		1.82(XI)	2.05(IX)		
Σexpected	3	2	2		

### Table 3 Comparison of the cell parameters of the three isostructural heptafluorides showing a noticeable anomaly on the *c* parameter of the chromium compound.

**Table:**

Formula	*a*	*b*	*c*	β	*V*
Pb_2_RhF_7_*^a^*	5.569	11.854	8.832	91.00	582.96
Sr_2_RhF_7_*^b^*	5.510	11.628	8.640	90.98	553.49
Pb_2_CrF_7_*^c^*	5.463	11.208	9.574	91.20	586.05

Notes: (*a*) Domesle & Hoppe (1983); (*b*) Grosse & Hoppe (1987); (*c*) this work.

## References

[bb1] Brandenburg, K. (2005). *DIAMOND* Crystal Impact GbR, Bonn, Germany.

[bb2] Brese, N. E. & O’Keeffe, M. (1991). *Acta Cryst.* B**47**, 192–197.

[bb3] Brown, I. D. & Altermatt, D. (1985). *Acta Cryst.* B**41**, 244–247.

[bb4] Domesle, R. & Hoppe, R. (1980). *Z. Kristallogr* **153**, 317–328.

[bb5] Domesle, R. & Hoppe, R. (1983). *Z. Anorg. Allg. Chem* **501**, 102–110.

[bb6] Farrugia, L. J. (1997). *J. Appl. Cryst.* **30**, 565.

[bb7] Grosse, L. & Hoppe, R. (1987). *Z. Anorg. Allg. Chem* **552**, 123–131.

[bb8] Kozak, A. de (1999). ICDD PDF card 00-051-0237.

[bb9] Le Bail, A. (1989). *J. Solid State Chem* **83**, 267–271.

[bb10] Le Bail, A. (1996). *Eur. J. Solid State Inorg. Chem.* **33**, 1211–1222.

[bb11] Le Bail, A. (2000). *J. Non-Cryst. Solids ***271**, 249–259.

[bb12] Le Bail, A. & Laval, J.-P. (1998). *Eur. J. Solid State Inorg. Chem* **35**, 357–372.

[bb13] Le Bail, A. & Mercier, A.-M. (1992). *Eur. J. Solid State Inorg. Chem* **29**, 183–190.

[bb14] Le Bail, A., Mercier, A.-M. & Dix, I. (2009). *Acta Cryst.* E**65**, i46–i47. 10.1107/S1600536809019126 PMC2969795 21582981

[bb15] Sheldrick, G. M. (2008). *Acta Cryst.* A**64**, 112–122. 10.1107/S0108767307043930 18156677

[bb16] Stoe & Cie (1998). *STADI4* and *X-RED* Stoe & Cie, Darmstadt, Germany.

[bb17] Westrip, S. P. (2010). *publCIF* In preparation.

